# Lower *PRDM2* expression is associated with dopamine-agonist resistance and tumor recurrence in prolactinomas

**DOI:** 10.1186/s12885-015-1267-0

**Published:** 2015-04-12

**Authors:** Hua Gao, Fei Wang, Xiaolei Lan, Chuzhong Li, Jie Feng, Jiwei Bai, Lei Cao, Songbai Gui, Lichuan Hong, Yazhuo Zhang

**Affiliations:** 1 Beijing Neurosurgical Institute, Capital Medical University, Beijing, China; 2 Department of Neurosurgery, Provincial Hospital Affiliated to Anhui Medical University, Hefei, Anhui Province China; 3 Neurosurgical Department, The Affiliated Hospital of Medical College, Qingdao University, China; and Capital Medical University, Beijing, China; 4 Neurosurgical Department of Beijing Tiantan Hospital, Beijing, China; 5 Tsinghua University, Beijing, China

**Keywords:** Prolactinomas, Whole-exome sequencing, Dopamine agonists, Drug resistance, PRDM2

## Abstract

**Background:**

Dopamine agonists (DAs) are the first-line treatment for prolactinomas, which account for 25–30% of functioning pituitary adenomas, and bromocriptine (BRC) is the only commercially available DAs in China. However, tumors are resistant to therapy in 5–18% of patients.

**Methods:**

The exomes of six responsive prolactinomas and six resistant prolactinomas were analyzed by whole-exome sequencing.

**Results:**

Using stringent variant calling and filtering parameters, ten somatic variants that were mainly associated with DNA repair or protein metabolic processes were identified. New resistant variants were identified in multiple genes including *PRDM2, PRG4, MUC4, DSPP, DPCR1, RP1L1, MX2, POTEF, C1orf170,* and *KRTAP10-3*. The expression of these genes was then quantified by real-time reverse-transcription PCR (RT–qPCR) in 12 prolactinomas and 3 normal pituitary glands. The mRNA levels of PRDM2 were approximately five-fold lower in resistant prolactinomas than in responsive tumors (p < 0.05). PRDM2 protein levels were lower in resistant prolactinomas than in responsive tumors, as determined by Western blotting and immunohistochemical analysis (p < 0.05). Overexpression of PRDM2 upregulated dopamine receptor D2 (D2DR) and inhibited the phosphorylation of ERK1/2 in MMQ cells. PRDM2 showed a synergistic effect with BRC on the inhibition of prolactin (PRL) secretion and MMQ cell viability, and low PRDM2 expression was associated with tumor recurrence.

**Conclusions:**

PRDM2 downregulation may play a role in dopamine-agonist resistance and tumor recurrence in prolactinomas.

**Electronic supplementary material:**

The online version of this article (doi:10.1186/s12885-015-1267-0) contains supplementary material, which is available to authorized users.

## Background

Prolactinomas account for 25–30% of functioning pituitary tumors, with an estimated prevalence of 100 cases per 100,000 individuals [1-3]. Pharmacological intervention is the first-line treatment and involves the use of dopamine agonists (DAs) to reduce tumor size and prolactin level. BRC, the oldest drug for the medical treatment of prolactinomas, was introduced into clinical practice approximately 30 years ago [1,4] and is the only commercially available DAs in China. In 80–90% of patients with microprolactinomas and 70% of individuals with macroprolactinomas, BRC treatment controls hyperprolactinemia, restoring gonadal function and reducing tumor size. Side effects of BRC include nausea, dizziness (orthostatic hypotension), nasal stuffiness, difficulty concentrating, depression, psychosis, and peripheral vasospasm and so on [1].

Resistance to BRC, defined as the absence of normalization of prolactin (PRL) levels despite a 15 mg daily dose of BRC during at least 3 months, has been observed in 5–18% of prolactinomas according to the literature [3,5]. The mechanisms underlying BRC resistance of prolactinomas are not fully understood, although such resistance to BRC correlates with reduced binding to the D2 receptor subtype of dopamine, the major PRL inhibiting factor. Resistance to BRC therapy may involve defects in D2 dopamine receptor expression and possibly its posttranscriptional splicing [6,7]. The ability to predict the degree and extent of BRC resistance would enable the design of personalized treatment regimens. Lack of therapeutic response to BRC may be an indication for surgery or radiation therapy. In the present study, we used stringent variant calling and filtering parameters and identified 10 somatic mutations mainly associated with DNA repair or the protein metabolic process by using whole-exome sequencing in combination with homozygosity mapping in a comparative analysis of BRC-responsive and BRC-resistant prolactinomas. We describe a new candidate gene associated with BRC resistance in prolactinomas, *PRDM2*, and show that the protein and mRNA levels of *PRDM2* are lower in BRC-resistant prolactinomas than in BRC-responsive prolactinomas.

## Methods

### Patients and specimens

Samples from six BRC-responsive prolactinomas and six BRC-resistant prolactinomas were obtained from the biobank of Beijing Neurosurgical Institute. And these patients underwent endoscopic trans-sphenoidal surgery between December 2008 and January 2011 at Tiantan Hospital, Beijing, China. Informed consent was obtained from patients using protocols approved by IRB of Beijing Tiantan Hospital Affiliated to Capital Medical University (KY2013-015-2). Pituitary prolactinomas which were obtained from 12 patients (8 men and 4 women, mean age 37.4 years,range 15–63 years) without a family history of endocrine neoplasia were characterized on presurgical clinical and biochemical findings and morphological and immunohistochemical analysis of removed tissue samples (Table 1). Resistant tumors were defined as those from patients whose serum PRL levels remained abnormally high after at least 3 months of treatment with a daily dose of at least 15 mg BRC(1; 3). The tumors did not have features of atypia, and they constituted the discovery set of tumors for exome capture and DNA sequence analysis. Additional twenty-four pituitary prolactinomas, histologically confirmed, were obtained from 8 women and 16 men (mean age 61 years, range 17–71 years), and these constituted the validation set (Table 2).

**Table 1:** **Patients classification according to their response to BRC**

Patient number	Serum PRL levels (μg/ml)			Tumor size(cm)	Higher BRC dose (mg/day)	Months with higher BRC dose	Classification
	Before BRC	With BRC	Normal?				
1	928	3.9	YES	2	5	6	Responsive
2	4843	15	YES	2.5	2.5	10	Responsive
3	122.3	3.18	YES	1.8	2.5	3	Responsive
4	899	7.6	YES	4.5	7.5	6	Responsive
5	3117	9.2	YES	2.5	7.5	3	Responsive
6	3830	15.5	YES	2	5	8	Responsive
7	975	165	NO	3.5	15	4	Resistant
8	>6000	268	NO	3	15	5	Resistant
9	182	83.2	NO	2	15	5	Resistant
10	>6000	68.2	NO	2.5	15	6	Resistant
11	2899	128.6	NO	5	15	24	Resistant
12	168	150	NO	0.8	15	6	Resistant

**Table 2:** **Relationship between** ***PRDM2*** **mRNA levels in prolactinomas and various clinical parameters**

	*PRDM2*mRNA levels			
Feature	High*	Low	Chi-square	*p*-Value
All cases	12	12		
Patient’s age				
≥50	5	6	0.168	0.682
<50	7	6		
Patient’s gender				
Male	9	7	0.756	0.385
Female	3	5		
Serum PRL levels				
≥435 μg/ml **	4	5	0.178	0.673
<435 μg/ml	8	7		
Tumor size				
≥2 cm	5	7	0.670	0.413
<2 cm	7	5		
Recurrence***				
Yes	4	10	6.511	0.011
No	8	2		
Resistance to BRC****				
	4	9	4.332	0.037
	8	3		

*The median expression level was used as the cutoff. Low PRDM2 mRNA levels were defined as values below the 50th percentile of the 12 patients; values at or above the 50th percentile were classified as high levels.

**The median serum PRL level was used as the cutoff: 435 μg/ml. Low serum PRL levels were defined as values below the 50th percentile of the 12 patients; values at or above the 50th percentile were classified as high levels.

***Recurrence was defined as the discovery of an elevated PRL level at any time in the postoperative surveillance period after an initial remission.

****Resistant tumors to BRC were defined as those from patients whose serum PRL levels remained abnormally high after at least 3 months of treatment with a daily dose of 15 mg BRC.

### Exome capture, DNA sequencing, and bioinformatics analysis

Total DNA was extracted from prolactinomas using the QIAamp DNA Mini Kit (QIAGEN, Hilden, Germany). An aliquot containing 5 μg of genomic DNA was purified and quantified from each specimen. Exome enrichment was performed by using an ABI SOLiD optimized SureSelect Human All Exon kit (Agilent, Santa Clara, CA, USA), which included the exonic sequences of ~18,000 genes, covering a total of 37 Mb of genomic sequences. The enriched exome libraries were then amplified by emulsion PCR(ePCR), according to the manufacturer’s instructions (Life Technologies, Carlsbad, CA, USA), based on a library concentration of 0.5 pM. The PCR products were then sequenced on a SOLiD5500 sequencer (Life Technologies); one quad of a SOLiD sequencing slide was required for each sample.

Color-space reads were mapped to the hg19 reference human genome using SOLiDBioScope software (Life Technologies), which is suitable for a repetitive mapping approach. Single-nucleotide polymorphisms (SNPs) were then called using the diBayes algorithm with conservative default call stringency [8]. We excluded known SNPs available from the Single Nucleotide Polymorphism Database (dbSNP) v130, maintained by the National Center for Biotechnology Information (NCBI).

### RNA extraction and real-time reverse-transcription quantitive PCR (RT–qPCR)

Total RNA was extracted from prolactinomas (~50 mg) stored in liquid nitrogen using the TRIzol Reagent (Life Technologies). The primers used in real-time reverse-transcription PCR (RT-qPCR) are listed in (Table 3). RT-qPCR was performed as described previously [3], using Applied Bio-systems 7500 Fast System (Life Technologies). The fold-change in differential expression for each gene was calculated using the comparative C_T_ method (also known as the 2^−∆∆CT^ method) as previously described [9].

**Table 3:** **Primers used for RT–qPCR analysis of the expression of 10 variant genes**

Gene	Forward primer	Reverse primer
*C1orf170*	5′-CACCCTGCGTTTCTTCTGG-3′	5′-TGCCCATCCCCTCTTTG-3′
*DPCR1*	5′-AGTGCTGCCTCCTCTTCCTTCTA-3′	5′-GGGAGCTCTGGAGGTCTTTGTC-3′
*DSPP*	5′-GCATTTGGGCAGTAGCATGG-3′	5′-CTGACACATTTGATCTTGCTAGGAG-3′
*KRTAP10-3*	5′-AGCCAGCTTGCTGCACAT-3′	5′-TGAAGAGGAAGCCCCAGAG-3′
*MUC4*	5′-GCCAACTTCACGCTCAGAGAC-3′	5′-TCTCCAGAGTGAATGGCTCCAG-3′
*MX2*	5′-GCCAGGTGGAGAAAGAGATACACAA-3′	5′-AGGTCAATGATGGTCAGGTCTGG-3′
*POTEF*	5′-CTGCATGTGGCGTGACTCTG-3′	5′-CGGCATGGAATCAACCTCAA-3′
*PRDM2*	5′-AGCAGCTGCGATTGAGGA-3′	5′-CAGAGGTGAAATCTGGCTCACTT-3′
*PRG4*	5′-GGCAGCGCTTTCAACAGCTAA-3′	5′-CCAGGGCACTTCTGTACAGGTTC-3′
*RP1L1*	5′-AGAAGCGAGGCTGAAACTTTATCTG-3′	5′-TCACACTCGGCTTGGTCTTTG-3′

### Plasmid constructs

The human PRDM2 cDNA was obtained by reverse transcriptase-polymerase chain reaction from human brain RNA with primers (forward 5’-TCTGCTGTTGACAAGACCC-3’, reverse 5’-GCATCAATGCACATCCATC -3’) designed according to the prdm2 sequence published in GenBank (NM_001135610.1). The cDNA was directionally cloned into the pCMV6 plasmid (Invitrogen). The orientation of the WT cDNA was verified by DNA sequencing.

### Assessment of cell viability

To evaluate the effect of PRDM2 on cell viability, 1 × 10^4^ MMQ cells were transfected with PRDM2 for 24 h, 48 h and 72 h in 96-well plates. A total of 20 μl of 5 mg/ml 3-(4,5-dimethylthiazol-2-yl)-2,5-diphenyl tetrazolium (MTT) was added into each well to continue the culture for another 4 h. Culture medium was discarded and dimethyl sulfoxide was added at 100 μl/well. The mixture was shaken for 10 min at a constant temperature of 37 C before absorbance measurement.

### Concentration of cell PRL secretion assays using ELISA

To evaluate the effect of PRDM2 on PRL secretion, 1 × 10^6^ MMQ cells were transfected with PRDM2 (1 μg) for 24 h. Addition of BRC (40nM) to test wells and PBS with corresponding solvent added to control wells was followed by culture for 24 h and centrifugation to collect the supernatant. Then following the directions of the rat PRL ELISA kit manual, the concentrations of PRL were estimated from a standard curve of known PRL concentrations.

### Immunohistochemical analysis of prolactinoma specimens

The immunohistochemical SABC method was used as described previously [8]. Prolactinoma and pituitary gland specimens were sectioned to a thickness of 5 μm in paraffin wax. The sections were subjected to gradient dewaxing, removed to water, treated with fresh 3% hydrogen peroxide at room temperature for 10 min, and washed with PBS (PH 7.2) 3 times for 5 min each. For microwave repair, the specimens were placed in 0.01% citric acid (pH 6.0), kept warm in a microwave oven (600 W) for 10 min, allowed to cool to room temperature, and washed once with PBS. Antibody repair solution I was added at room temperature for 10 min, and then washed 3 times with PBS for 5 min each time. Primary PRDM2 (RIZ1) antibody was added at a 1:200 dilution and incubated at 4°C overnight. The daylight™ conjugated affinipure second antibody with fluorescence was added at room temperature for 1 h followed by 3 washes with PBS for 5 min each time. SABC was added for 20 min and then washed with adequate PBS. Sections were mounted with Prolong Gold Antifade reagent with 4′,6-diamidino-2-phenylindole (DAPI) (Invitrogen). Sections were analyzed with a LEICA-TCS-SP5II to estimate the percentage of DAPI-stained cells displaying PRDM2 immunoreactivity.

### SDS–PAGE and Western blot analyses

Prolactinomas and normal pituitary specimens were lysed in TNE buffer (50 mM Tris–HCl, pH 7.4, 150 mM NaCl, 1 mM EDTA; all from Sigma-Aldrich) containing 1% Nonidet P-40 (Calbiochem) with protease and phosphatase inhibitor cocktails (Roche). Total extracts were centrifuged at 12000 × *g* for 30 min at 4°C, and the protein concentration of the supernatant was determined with a BCA protein assay kit (Pierce Biotechnology). For western blot analysis, 40 μg of lysate per lane was loaded onto 4–12% Bis-Tris SDS-PAGE gels, separated electrophoretically, and blotted onto polyvinylidene fluoride (PVDF) membranes. Different blots were incubated with antibodies against D2DR (1:2000, Abcam), p-ERK1/2 (1:1000, CST), PRL (1:1000, Santa-Cruz), T-ERK1/2(1:1000, CST), PRDM2 (1:500, Abcam) and β-actin (Sigma) followed by secondary antibodies tagged with horseradish peroxidase (Santa Cruz Biotechnology). Blots were visualized by enhanced chemiluminescence, and densitometry was performed with a Versadoc XL imaging apparatus (Bio-Rad). Analysis of β-actin levels was used as a loading control.

### Statistical analysis

All the statistical analyses were performed using SPSS version 20.0. For comparisons, one-way analyses of variance, chi-squared tests, Wilcoxon rank-sum test and two-tailed Student’s *t*-tests, were carried out as appropriate. Binary logistic regression was performed to identify independent factors related to prolactinoma recurrence.

## Results

### Identification of variant genes by whole-exome sequencing

The exomes of six BRC-responsive prolactinomas and six BRC-resistant prolactinomas were analyzed by whole-exome sequencing which yielded excellent target region coverage with approximately 71% of the exome covered to a depth of at least ten-fold between the somatic variant calling algorithm and confirmatory sequencing. Several prioritization steps were taken to decrease the number of genetic variants and to find the potentially pathogenic variants [10]. Approximately 90% of single-nucleotide variants (SNVs) resulted in missense amino-acid changes, whereas the remaining approximately 10% were synonymous changes. More than 70% of the SNVs occurred as C: G–T: A transitions, and less than 30% were transversions. Using stringent variant calling and filtering parameters, 119 somatic variants were identified in these specimens. A comparison with the NCBI dbSNP, with recently released SNP data from other groups and with in-house SNP data confirmed that >90% of the identified variants were previously reported SNPs that did not seem to explain BRC resistance of prolactinomas. Ten variant genes were selected for further study: *C1orf170*, *DPCR1*, *DSPP*, *KRTAP10-3*, *POTEF*, *MUC4*, *MX2*, *PRDM2*, *PRG4*, *RP1L1* (Table 4) and the original data were shown in Additional file 1: Table S1.

**Table 4:** **Genes potentially related to BRC resistance in prolactinomas**

Gene	GenBank ID	Encoded protein
*C1orf170*	BC006300	Uncharacterized protein, chromosome 1 ORF 170
*DPCR1*	NM_080870	Diffuse panbronchiolitis critical region protein 1
*DSPP*	NM_014208	Dentin sialophosphoprotein
*KRTAP10-3*	NM_198696	Keratin associated protein 10-3
*POTEF*	NM_001099771.2	POTE ankyrin domain family, member F
*MUC4*	NM_138297.4	Mucin 4, cell surface associated
*MX2*	NM_002463	Myxovirus (influenza virus) resistance protein 2
*PRDM2*	NM_001007257.2	PR domain containing 2, with ZNF domain
*PRG4*	NM_005807.	Proteoglycan 4 (PRG4), transcript variant A
*RP1L1*	NM_178857	Retinitis pigmentosa 1-like 1 protein

### Analysis of the expression of variant genes by RT–qPCR

**Figure 1:**
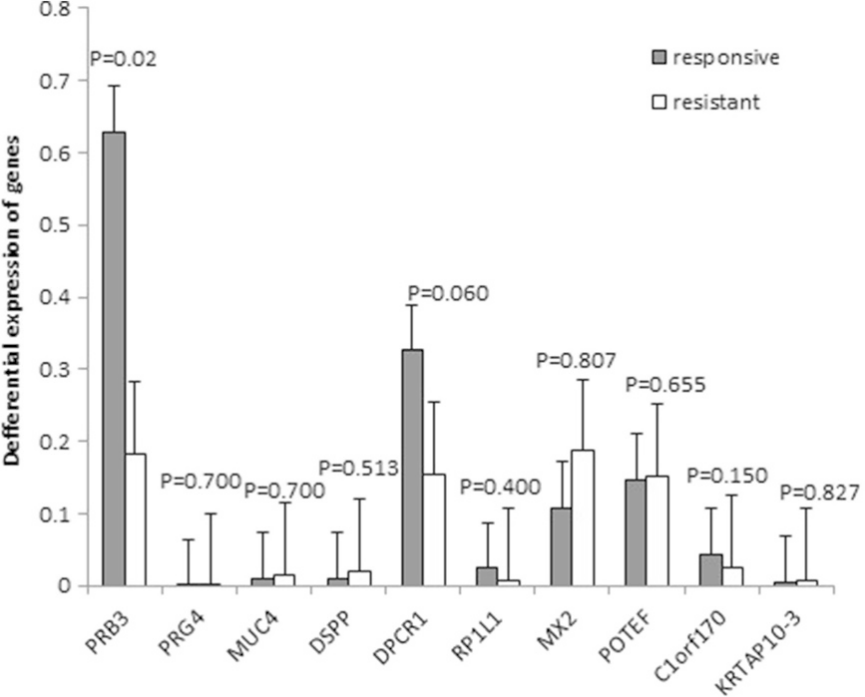
RT-qPCR analysis of 10 variant genes in BRC-responsive and BRC-resistant prolactinomas. Only the differential expression of PRDM2 was statistically significant.

We used real time quantitative PCR (RT–qPCR) to test whether BRC resistance in prolactinomas was associated with differences in the expression levels of any of 10 variant genes. Indeed, the mRNA levels of *C1orf170*, *DPCR1*, *KRTAP10-3*, *PRDM2* and *RP1L1* were 50% lower in resistant tumors than in responsive tumors. In particular, PRDM2 mRNA levels were approximately five-fold lower in resistant prolactinomas than in the responsive tumors (p < 0.05). By contrast, the mRNA levels of *DSPP*, *PRG4*, *MUC4*, *POTEF* and *MX2* were higher in resistant prolactinomas than in the responsive tumors (p > 0.05). Mean expression levels of the 10 genes are shown in Figure 1 and Table 5 (statistical method: two-tailed Student’s t tests).

**Table 5:** **Differential expression folds of genes**

	Responsive	Resistant	p value
PRDM2	0.260138247	0.05833261	0.020
PRG4	0.000109963	0.000281276	0.700
MUC4	0.010466577	0.015666494	0.700
DSPP	0.010298289	0.021137023	0.513
DPCR1	0.3257084	0.154926532	0.060
RP1L1	0.023944092	0.006293673	0.400
MX2	0.108311332	0.18653675	0.807
POTEF	0.146750534	0.150689894	0.655
C1orf170	0.043853399	0.024682049	0.150
KRTAP10-3	0.003738342	0.006845497	0.827

### Identification of candidate driver mutations

Somatic mutations in genes previously associated with pituitary tumorigenesis including *AIP*, *BMP-4*, *CDKN1B*, *CDKN2A*, *CDKN2C*, *Cyclin D1*, *D2R*, *GADD45G*, *Gsp*, *MEG3a*, *MEN1*, *p53*, *Pdt-FGFR4*, *PKC*, *PRKAR1A*, *PTTG*, *RAS*, *SSTR2/SSTR5*, *WIF-1* and *ZAC1*[8,11] were not detected. To identify putative driver mutations, further analysis of each of the somatic variants was performed. We hypothesized that putative driver mutations would typically: 1) have a deleterious effect on protein function; 2) be present at sufficient allele frequency to represent likely heterozygous or homozygous changes; and 3) be involved in biological processes relevant to tumorigenesis [12].

**Figure 2:**
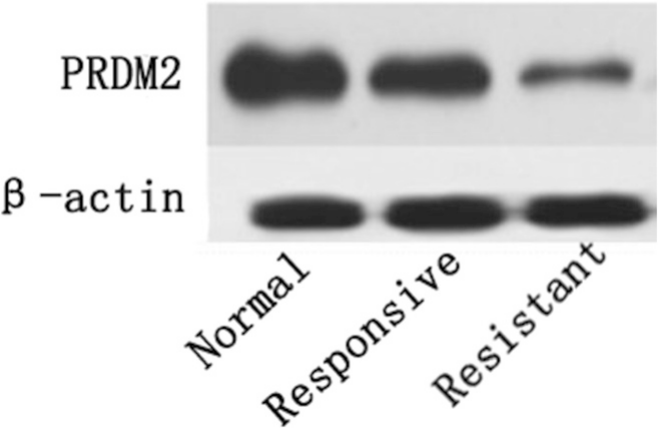
The protein level of RIZ1 in groups.

**Figure 3:**
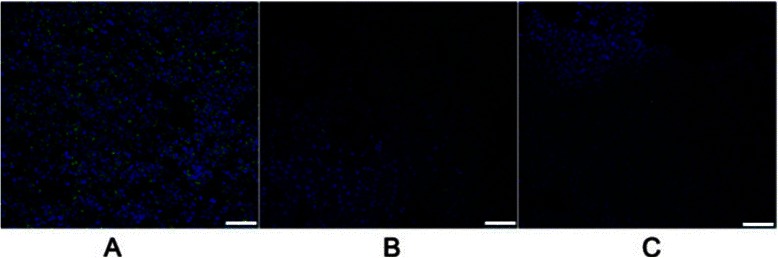
The expression level of RIZ1 in different groups through Confocal. **A**: Normal. **B**: BRC-Responsive prolactinomas; **C** BRC-Resistant prolactinomas. Green: RIZ1 Blue: DAPI. Bar: 50 μM.

**Figure 4:**
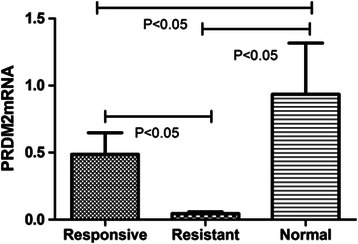
Mean PRDM2 mRNA levels in BRC-responsive and BRC resistant prolactinomas and in normal pituitary. PRDM2 expression was measured by RT-qPCR in 6 BRC-responsive prolactinomas, 6 BRC resistant prolactinomas and 6 normal pituitary glands. Horizontal lines above the bars represent standard deviations (statistical method: one-way analyses of variance).

Using these criteria, *PRDM2* (retinoblastoma interacting zinc-finger protein, RIZ) was selected for further evaluation. The *PRDM2* gene was isolated in a functional screening for Rb-binding proteins. The *PRDM2* gene is one of the candidate tumor suppressor genes on chromosome 1p. Moreover, inactivation of the *PRDM2* gene by promoter hypermethylation has been reported in breast, liver, and gastric carcinomas [13,14]. To investigate the potential role of *PRDM2* in BRC resistance, we measured the level of PRDM2 expression by western blotting and immunohistochemistry in six resistant prolactinomas, six sensitive prolactinomas and three normal pituitary glands. The PRDM2 protein level in the BRC-resistant group was approximately 24.6 ± 5.2% of that in the normal pituitary gland (p < 0.01); however, the PRDM2 protein level in the BRC-responsive group was 78.3 ± 6.1% of that in the normal pituitary gland (p < 0.05) (Figure 2). PRDM2 protein levels were obviously lower in resistant prolactinomas than in the responsive tumors (p < 0.05). Confocal images showed that the number of PRDM2-positive puncta in BRC-resistant prolactinomas was lower than that of the normal pituitary and sensitive prolactinomas (Figure 3). And the levels of *PRDM2* mRNA in BRC-resistant prolactinomas were about five lower than in the responsive tumors (p < 0.05), and about eight fold lower than in normal pituitary glands (Figure 4).

### PRDM2 showed a synergistic effect with BRC on the inhibition of PRL secretion and MMQ cell viability

**Figure 5:**
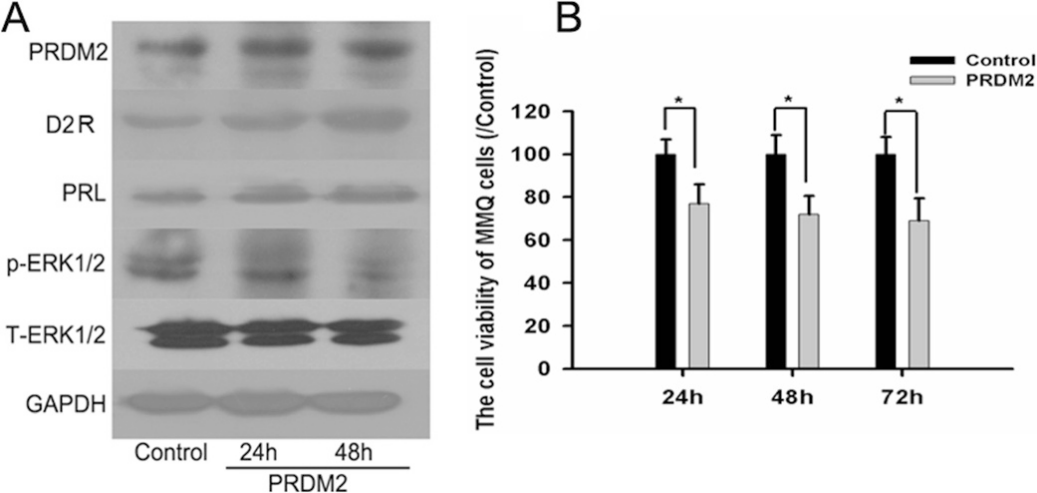
Overexpression PRDM2 could increase the D2R level and inhibit the cell proliferatin of MMQ cells. **A**: Overexpression PRDM2 could increase the D2R level and reduce the phosphorylation of ERK1/2 in MMQ cells. **B**: Overexpression of PRDM2 could inhibit the cell proliferation of MMQ cells. n = 3 * compared to Control group.

BRC is derived by semisynthesis and has D2 receptor agonist and D1 receptor antagonist properties. We found that overexpression of PRDM2 could increase the D2R level and PRL level of MMQ cells by Western blot (Figure 5A). In additional, PRDM2 could reduce the phosphorylation of ERK1/2 (Figure 5A) and reduce the cell viability of MMQ cells (Figure 5B).

**Figure 6:**
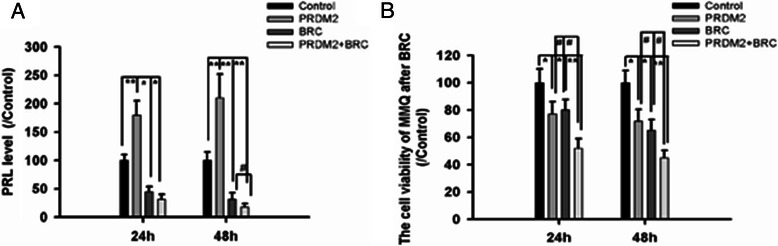
PRDM2 showed a synergy effect with BRC on the inhibition of PRL secretion and MMQ cell viability. **A**: The PRL level of MMQ cells after PRDM2 and BRC. *compared to Control group # compared to BRC group **B**: The cell viability of MMQ cells after PRDM2 and BRC. *compared to Control group # compared to PRDM2 group.

To evaluate the effect of PRDM2 on PRL secretion, 1 × 10^6^ MMQ cells were transfected with PRDM2 (1 μg) for 24 h. Another 24 h was cultured with BRC (40nM) to test wells and isometric DMSO as control wells. BRC could inhibit the PRL secretion and MMQ cell viability. We found that PRDM2 showed a synergy inhibition with BRC on PRL secretion and MMQ cell viability (Figure 6).

### Low PRDM2 mRNA levels is associated with tumor recurrence in prolactinomas

Between 2008 and 2013, 24 patients with prolactinomas were enrolled in our study. Follow-up periods ranged from 6 months to 5 years (mean 4.0 years). The median expression level was used as the cutoff. Low PRDM2 mRNA levels were defined as values below the 50th percentile of the 12 patients; values at or above the 50th percentile were classified as high levels. We then asked whether low PRDM2 mRNA levels in prolactinomas were associated with any clinical parameters (Table 2.) (Chi squared tests). There was no significant correlation between PRDM2 mRNA levels and age, gender, tumor size or PRL serum levels. However, low PRDM2 mRNA levels were more frequently observed in recurrent tumors (p = 0.011) and BRC-resistant tumors (p = 0.037). Recurrence was defined as the discovery of an elevated PRL level at any time in the postoperative surveillance period after an initial remission [1]. Furthermore, binary multivariate regression revealed that low levels of PRDM2 mRNA were independently associated with tumor recurrence (odd ratio [OR] 0.065, 95% confidence interval [CI]: 0.05–0.832, p = 0.036).

## Discussion

Previous studies showed that some DA-resistant prolactinomas have reduced dopamine d2 receptor density using different methods [15,16]. The proportion of D2R-encoding mRNA corresponding to the D2S isoform was lower in resistant prolactinomas than in responsive tumors have demonstrated by other studies [17,18]. Whole-exome sequencing identified sequence variants associated with 10 genes, not previously implicated in DA-resistant prolactinomas. In addition, our analysis identified somatic variants in established oncogenes, tumor suppressor genes and genes associated with DNA repair and protein metabolic processes.

In the present study, PRDM2 mRNA levels were approximately five-fold lower in resistant prolactinomas than in the responsive tumors (p < 0.05). PRDM2 protein levels were significantly lower in resistant prolactinomas than in the responsive tumors (p < 0.05). In addition, Confocal images showed that the number of PRDM2-positive puncta in BRC-resistant prolactinomas was lower than that of the normal pituitary and sensitive prolactinomas (p < 0.05). We found that PRDM2 showed a synergy inhibition with BRC on PRL secretion and MMQ cell viability. Further analysis of our data confirmed that low levels of *PRDM2* were more frequently observed in recurrent tumors. Furthermore, binary multivariate regression analysis revealed that low *PRDM2* expression level was independently associated with prolactinoma recurrence (OR 0.065; 95% CI: 0.05–0.832; p = 0.036).

Pellegrini et al. [15] in 1989 found that D2R levels were lower in dopamine resistant prolactinomas. And then, the same group showed also a lower expression of pituitary specific PIT1 (POU1F1) transcription factor in dopamine resistant prolactinomas [18]. Raverot et al. [19] found already seven genes mRNA level variation, notably *PPTG* and *CCNB*1, were associated with tumor recurrence or progression. Delgrange et al. [20] reported that resistant prolactinomas tend to be more invasive and to recur more often than responsive tumors. Furthermore, recurrent prolactinomas were more likely to be resistant to the drug therapy. Some authors [3,18,21] reported that resistant prolactinomas tend to be more invasive and recur more often than responsive tumors. Furthermore, recurrent prolactinomas are more likely to be resistant to drug therapy.

Buyse et al. first found *PRDM2* by retinoblastoma (Rb) probes in 1995, while performing a functional screen for Rb, and observed that it was able to interact with Rb [22]. Fluorescent in situ hybridization showed that the *PRDM2* gene is located on human chromosome 1p36. There are two variants of *PRDM2, RIZ1* and *RIZ2*, according to two alternative initial locations [23,24]. The sequences of *RIZ1* and *RIZ2* are same, except the presence of a PR domain in RIZ1. Generally, RIZ1 is refers to PRDM2 protein. The PR domain in RIZ1 is named after the PRDI-BF1-RIZ1 region and contains about 100 amino acids. The role of the structure is to stabilize chromosomal structures and, therefore, mediate gene expression [25]. The presence or absence of a PR domain results in the differential expression of proteins at an early stage of tumorigenesis, which provides a mechanism for tumor initiation [26]. Some reports have demonstrated that RIZ1 is able to inhibit tumor development and is thought to be a tumor suppressor gene [14,27,28]. Furthermore, because RIZ1 closely interacts with Rb, which induces the arrest of tumor cells in the G2/M phase resulting to cell death, up-expression of RIZ1 may lead to arrest of tumor [26].

As a new tumor suppressor, *PRDM2* has been analyzed in a few reports, which have attempted to explain the reasons the gene is inactivated in cancer cells. Genetic and epigenetic changes are thought to be responsible [29,30]. According to a genetic perspective, *PRDM2* may be down-regulated by chromosomal instability and microsatellite instability, as well as frame-shift mutations, point mutations and heterozygote deficiency [14,29,31]. From an epigenetic perspective, the deactivation of *PRDM2* may occur due to promoter methylation and histone acetylation [32]. Changes to chromosome 1p36, on which *PRDM2* is located, are also associated with numerous types of cancer, including breast cancer, ovarian cancer, liver cancer, colorectal cancer, chronic myeloid leukemia, melanoma, chromaffin tumor and neuroblastoma [29]. In all these tumor types, the tumor tissue samples and cancer cell lines displayed low expression levels or deficiency of PR*DM2*. In our study, we found the low *PRDM2* level in BRC-resistance prolactinomas because of inser mutation (chr1:14106398, TCC). Overexpression of *PRDM2* in MMQ cells could up-regulate the D2DR lelvel, and we speculates that *PRDM2* is potential synergy inhibition with BRC through the PRL secretion and cell viability tests of MMQ cells.

BRC inhibits DNA synthesis and delays the cell cycle [33]. The BRC-resistance of prolactinomas is associated with several processes including drug efflux by transporters, inactivation by detoxifying enzymes, altered expression of pro-/anti-apoptotic proteins or tumor suppressors, and increased activity of DNA repair mechanisms [34,35]. In many cases, only single base pair changes are required to activate, silence, or functionally alter critical genes (e.g., proto-oncogenes, tumor suppressor genes) in a cell type-, differentiation stage-, and carcinogen-specific manner. The DNA repair machinery can be seen as a “caretaker” [36] in charge of preserving the integrity and stability of the genome. Specific base substitutions (point variants) in genomic DNA may be caused by endogenous and exogenous mechanisms and DNA-reactive agents. In the present study, 10 variants were associated with DNA repair or DNA metabolic processes, including *PRDM2, PRG4, MUC4, DSPP, DPCR1,* etc. Frame-shift mutations of the *PRDM2* gene are also common in microsatellite instability-positive tumors and truncate a PR-interacting domain [23].

## Conclusion

In summary, our results suggest that low levels of PRDM2 may contribute to promote drug resistance and tumor recurrence of prolactinomas in an as yet unknown manner. Further work will be required to extend the potential links between *PRDM2*, drug resistance and tumor recurrence. Our results demonstrate that whole-exome sequencing will be particularly valuable for gene discovery under conditions in which mapping has been confounded by locus heterogeneity and uncertainty about the boundaries of diagnostic classification, pointing to a bright future for its broad application to medicine.

## Additional file

Additional file 1: Table S1. The original data of 10 genes with mutations in prolactinomas.

## Abbreviations

MTT: 3-(4,5-dimethylthiazol-2-yl)-2,5-diphenyl tetrazolium
CI: Confidence interval
DAPI: 4′,6-diamidino-2-phenylindole
DA: Dopamine agonists
BRC: Bromocriptine
DNA: Deoxyribonucleic acid
D2DR: Dopamine receptor D2
NCBI: National Center for Biotechnology Information
PRL: Prolactin
RT–qPCR: Real-time reverse-transcription PCR
RNA: Ribonucleic acid
SNP: Single-nucleotide polymorphism
TNE: Tris sodium chloride EDTA buffer
PVDF: Polyvinylidene fluoride
RIZ: Retinoblastoma interacting zinc-finger protein
