# Supplementary figures and images for: Lower PRDM2 expression is associated with dopamine-agonist resistance and tumor recurrence in prolactinomas
Source: BMC Cancer. 2015 Apr 12;15:272. doi: 10.1186/s12885-015-1267-0 (PMC4407330; doi:10.1186/s12885-015-1267-0)

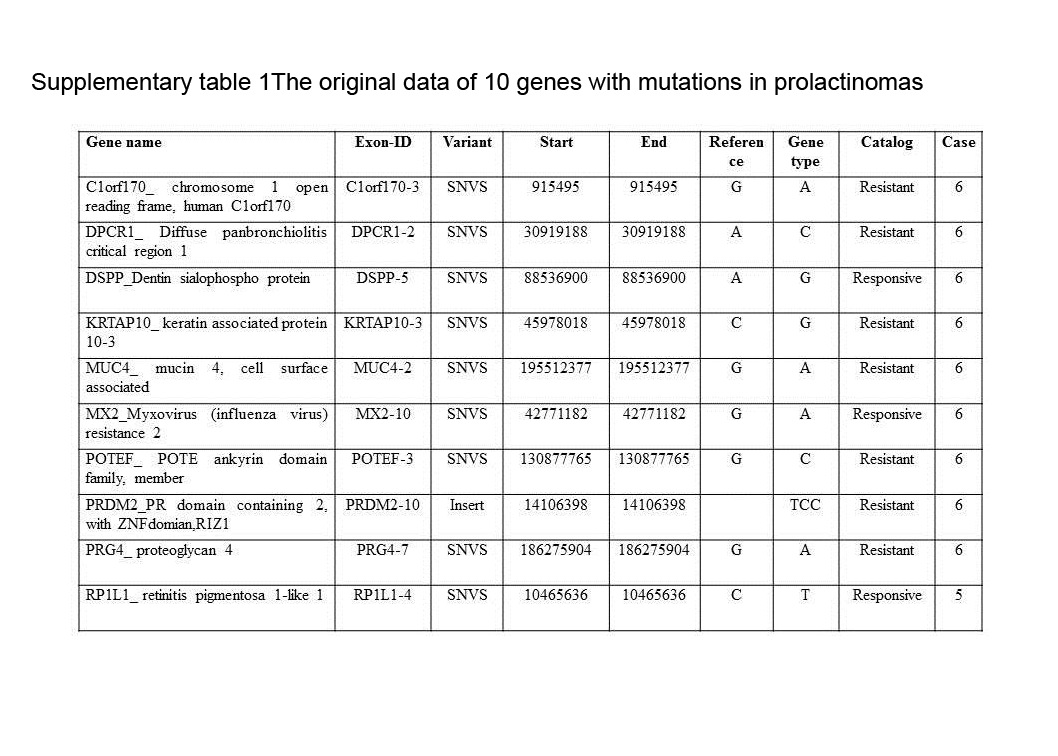

Supplement: Additional file 1: Table S1. — The original data of 10 genes with mutations in prolactinomas. [file 12885_2015_1267_MOESM1_ESM.tiff]
